# Evaluating serum procollagen type III N-terminal peptide use as a liver fibrosis screening test in patients with psoriasis receiving methotrexate: a retrospective service evaluation in comparison with the Fibrosis-4 (FIB-4) index

**DOI:** 10.1093/skinhd/vzag116

**Published:** 2026-07-31

**Authors:** Gary Roulston, Conall McKinney, Maia Monaghan, Catriona McMullan, Kathryn Ryan, Derek McKillop, David Alderdice

**Affiliations:** Department of Clinical Biochemistry, South Eastern Health and Social Care Trust, Ulster Hospital, Belfast, UK; Department of Dermatology, South Eastern Health and Social Care Trust, Ulster Hospital, Belfast, UK; Department of Dermatology, South Eastern Health and Social Care Trust, Ulster Hospital, Belfast, UK; Department of Dermatology, South Eastern Health and Social Care Trust, Ulster Hospital, Belfast, UK; Department of Clinical Biochemistry, South Eastern Health and Social Care Trust, Ulster Hospital, Belfast, UK; Department of Clinical Biochemistry, South Eastern Health and Social Care Trust, Ulster Hospital, Belfast, UK; Department of Dermatology, South Eastern Health and Social Care Trust, Ulster Hospital, Belfast, UK

## Abstract

Whilst procollagen type III N-terminal peptide (PIIINP) is recommended as a marker of liver fibrosis in patients with psoriasis on methotrexate, its efficacy is increasingly questioned. PIIINP is not specific as a marker of liver fibrosis, is expensive and has a lengthy turnaround time. There is growing evidence that methotrexate is not a direct cause of liver disease. Fibrosis-4 (FIB-4) is a validated screening score of liver fibrosis that has a substantial weight of evidence. FIB-4 scores are quick to calculate, minimally invasive and cost-effective. Our study reviews PIIINP use versus FIB-4 use in patients with psoriasis about to start or taking methotrexate therapy. This service evaluation demonstrates poor real-world adherence to PIIINP monitoring guidance and a lack of correlation between PIIINP and FIB-4 referral thresholds.

Dear Editor, Procollagen III N-terminal peptide (PIIINP) is released during the synthesis and deposition of type III collagen. Measurement of serum PIIINP as a marker of liver fibrosis has been recommended by the British Association of Dermatologists (BAD) in patients with psoriasis receiving methotrexate, both before treatment and at 3-monthly intervals.^1^ This recommendation was based on observational data and large case series, prior to a growing body of ­evidence suggesting that methotrexate is not a direct cause of liver ­fibrosis.^2,3^ Recent studies have suggested that cumulative methotrexate dosing is not associated with liver fibrosis in patients with ­psoriasis, with metabolic liver disease or other aetiologies likely to account for most liver abnormalities observed.

Hepatology guidelines have increasingly advocated for non­invasive fibrosis scores, such as the Fibrosis-4 (FIB-4) index, for risk stratification in patients with metabolic dysfunction-­associated steatotic liver disease (MASLD).^4,5^ International dermatology guidelines also recognize FIB-4 as an appropriate ­screening tool in patients at risk of fibrosis.^6^

PIIINP is relatively costly, mostly available as a referral test to specialist laboratories and has a long turnaround time. In contrast, FIB-4 incorporates routine laboratory tests [platelets, aspartate aminotransferase (AST) and alanine aminotransferase (ALT)] and age, making it a rapid and relatively inexpensive alternative. We therefore conducted a retrospective service evaluation to assess real-world PIIINP use in comparison with BAD guidance. We also compared PIIINP-based referral thresholds with retrospectively calculated FIB-4 scores. This evaluation was not designed as a diagnostic accuracy study.

As per BAD guidelines, PIIINP will only be requested in:

- Patients with psoriasis AND;
- Starting on, or already on, methotrexate AND;
- PIIINP every 3 months as part of methotrexate monitoring.
- Patients will be referred to hepatology for further investigations if:
  - PIIINP is >8 mg L^–1^ on two occasions; OR
  - three measurements are >4.2 mg L^–1^ in a 12-month period; OR
  - >10 mg L^–1^ on one occasion.

This retrospective service evaluation was conducted at a UK healthcare trust. Adult patients with psoriasis who had serum PIIINP requested between November 2023 and February 2025 were included. Encounters related to non-psoriasis diagnoses were excluded from subgroup analysis.

Data were collected by four data collectors (one chemical pathologist, three dermatologists). Clinical case notes were reviewed for demographics, methotrexate exposure, indication for methotrexate, liver biochemistry, timing of PIIINP measurements, referral decisions and hepatology review outcomes. FIB-4 scores were calculated retrospectively where platelet count, AST and ALT were available. When hepatology review occurred, fibrosis assessment was performed based on standard clinical pathways and included transient elastography (fibroscan). Liver biopsy was not performed in any case.

A total of 212 samples were sent for PIIINP from 169 patients. All were requested by dermatologists. Of these clinical encounters, 73.1% (*n* = 155/212) patients had psoriasis. The next most common diagnosis was eczema at 22.6% (*n* = 48/212). The results from patients with psoriasis form the basis of this analysis. The 155 clinical encounters equated to 118 patients. Fifty-five per cent (*n* = 85/155) were receiving methotrexate and the remainder (45.2%; *n* = 70/155) were undergoing pretreatment assessment.

PIIINP was frequently used outside BAD guidance, including in patients with a non-psoriasis diagnosis, and at a median testing interval of 7 months. Only 3.4% (*n* = 4/118) of patients met the PIIINP referral criteria, of whom one was referred to hepatology (Table 1). A total of 7.6% (*n* = 9/118) met FIB-4 referral criteria, with only one referred to hepatology (Table 1).

**Table 1:** Patients with psoriasis meeting procollagen III N-terminal peptide (PIIINP) British Association of Dermatologists (BAD) referral criteria and Fibrosis-4 (FIB-4) referral criteria

	*n* (%)
Met PIIINP BAD referral criteria	
Were they referred to hepatology?	
Yes	1 (20)
No	5 (80)
Met FIB-4 referral criteria	
Yes	0 (0)
No	6 (100)
Abnormal transaminases	
Yes	0 (0)
No	6 (100)
On methotrexate	
Yes	4 (100)
No	0 (0)
Met PIIINP criteria prior to methotrexate	
Yes	1 (25)
Likely^a^	2 (50)
No	1 (25)
Met FIB-4 referral criteria	
Were they referred to hepatology?	
Yes	1 (11)
No	8 (89)
Met PIIINP BAD criteria	
Yes	0 (0)
No	9 (100)
Abnormal transaminases	
Yes	5 (56)
No	4 (44)
On methotrexate	
Yes	7 (78)
No	2 (22)
Met FIB-4 criteria prior to methotrexate	
Yes	4 (44)
No	3 (33)
Unable to calculate	2 (22)

^a^Raised PIIINP (>4.2 mg L^–1^ but <8 mg L^–1^) on <3 occasions prior to methotrexate initiation.

At PIIINP sample collection, 60 patients were receiving methotrexate and 58 patients were not. Seven (12%) receiving methotrexate had a FIB-4 score suggesting hepatology referral. Of those not receiving methotrexate at time of PIIINP, two (3%) would have met FIB-4 referral criteria. Four patients (7%) on methotrexate met the PIIINP referral criteria, compared with none who were not on methotrexate, although prior to methotrexate starting three would have probably or definitely met the PIIINP referral criteria.

Importantly, there was no overlap in the patients identified by the two referral criteria. The patients meeting PIIINP referral criteria did not meet the FIB-4 referral criteria and vice versa.

Three patients with psoriasis were referred to hepatology overall. All were found to have liver steatosis but not fibrosis. They were managed conservatively with lifestyle advice. Referral decisions appeared to reflect clinical judgement and were influenced by liver enzyme results and the overall clinical context rather than by strictly following isolated biomarker thresholds.

Given the descriptive nature of this service evaluation, the small number of referrals for fibrosis assessment and the absence of confirmed fibrosis, inferential statistical analysis was not performed.

This service evaluation demonstrates regular deviation from BAD guideline PIIINP monitoring recommendations and highlights substantial variability in real-world practice, including over a quarter of samples being checked in patients with a non-­psoriasis diagnosis. A key finding is the complete lack of overlap between patients meeting PIIINP and FIB-4 referral thresholds, potentially suggesting that these tools are identifying distinct patient groups rather than corroborating fibrosis risk. FIB-4 is more aligned with abnormal liver enzymes, whereas PIIINP may be identifying patients without significant biochemical evidence of liver dysfunction. This lack of overlap raises important questions about the use of PIIINP as marker of fibrosis in this population, particularly given its known lack of specificity and elevation in other fibrotic and inflammatory conditions. However, FIB-4 incorporates markers more directly linked to hepatic disease.

Of note, no patient developed confirmed liver fibrosis in this cohort. This is consistent with evidence that methotrexate is not an independent driver of fibrosis, with studies demonstrating the rarity of clinically significant fibrosis in patients treated long-term with methotrexate, and that most liver abnormalities in patients with psoriasis reflect metabolic liver disease.^2,3^ FIB-4 has a well-established evidence base in MASLD, where it is a widely recommended tool for fibrosis risk stratification,^4,5^ whereas the evidence supporting PIIINP is limited and increasingly questioned.^7,8^

This service evaluation study does not demonstrate diagnostic superiority of FIB-4 over PIIINP. Instead, it emphasizes the low diagnostic yield and limited clinical impact of routine PIIINP monitoring in clinical practice, particularly given its cost, prolonged turnaround time and poor adherence to clinical guideline use. The frequent ordering of PIIINP outside of psoriasis indications may reflect legacy practice or a misunderstanding of BAD guidance.^7^

Altogether, these findings support a move towards risk-­stratified, metabolically focused monitoring strategies in patients with psoriasis, aligning dermatology practice more closely with contemporary hepatology and international dermatology guidance. The established role of FIB-4 in MASLD may provide an alternative tool for risk assessment; however, further prospective studies are required to determine optimal surveillance pathways and validate alternative approaches within populations with psoriasis.

The strengths of this evaluation include the capturing of real-word clinical practice and a direct comparison of two commonly cited monitoring strategies.

Limitations include the retrospective design, its relatively short observation period of 16 months for a slowly progressive condition such as liver fibrosis, the small number of referrals to hepatology and a restriction to patients who had PIIINP testing. This may limit generalizability and preclude diagnostic accuracy analysis.

## Data Availability

The data underlying this article will be shared on reasonable request to the corresponding author.
